# Quantitative Trait Locus Mapping of Salt Tolerance and Identification of Salt-Tolerant Genes in *Brassica napus* L

**DOI:** 10.3389/fpls.2017.01000

**Published:** 2017-06-14

**Authors:** Lina Lang, Aixia Xu, Juan Ding, Yan Zhang, Na Zhao, Zhengshu Tian, Yaping Liu, Yang Wang, Xia Liu, Fenghao Liang, Bingbing Zhang, Mengfan Qin, Jazira Dalelhan, Zhen Huang

**Affiliations:** State Key Laboratory of Crop Stress Biology for Arid Areas, College of Agronomy, Northwest A&F UniversityYangling, China

**Keywords:** salt tolerance, QTLs, candidate genes, cloning, expression, *Brassica napus*

## Abstract

Salinity stress is one of typical abiotic stresses that seriously limit crop production. In this study, a genetic linkage map based on 532 molecular markers covering 1341.1 cM was constructed to identify the loci associated with salt tolerance in *Brassica napus*. Up to 45 quantitative trait loci (QTLs) for 10 indicators were identified in the F_2:3_ populations. These QTLs can account for 4.80–51.14% of the phenotypic variation. A major QTL, qSPAD5 on LG5 associated with chlorophyll can be detected in three replicates. Two intron polymorphic (IP) markers in this QTL region were developed successfully to narrow down the QTL location to a region of 390 kb. A salt tolerance related gene *Bra003640* was primary identified as the candidate gene in this region. The full length of the candidate gene was 1,063 bp containing three exons and two introns in *B. napus* L. The open reading frame (ORF) is 867 bp and encodes 287 amino acids. Three amino acid differences (34, 54, and 83) in the conserved domain (B-box) were identified. RT-qPCR analysis showed that the gene expression had significant difference between the two parents. The study laid great foundation for salt tolerance related gene mapping and cloning in *B. napus* L.

## Introduction

Soil salinization is one of the crises the world is facing today. Salty soil is a low-yield soil which is widely distributed in the world. It is reported that approximately 37 million hectares of land is exposed to the salinity and secondary salinization in China (Zhang et al., 2007). In recent years, a large number of rapeseed acreage has been reduced rapidly in China's Yangtze River Basin because of low profit, which aggravated the gap of China's edible oil (Yin et al., 2009). Making full use of the saline-alkali soil and planting rapeseed in the saline-alkali soil is one of the most effective ways to alleviate the gap of edible oil in China (Ma et al., 2001). However, the prerequisite for this approach is to cultivate salt-tolerant rapeseed varieties.

Salt tolerance of rapeseed is a quantitative trait, and its phenotype is difficult to be determined under the normal condition. Many complex mechanisms are involved at different plant developmental levels. Salt tolerance in plants is a developmentally regulated phenomenon and the tolerance at one growth stage sometimes may not correlate with that of other stages (Kumar et al., 2015), which has brought difficulties for the genetic research of salt tolerance. For one stage of plant growth, researchers often use some physiological and morphological indicators to indirectly determine salt tolerance. The effect of salt stress on plant growth is the result of mineral deficit, ion toxicity, and osmotic stress (Munns, 2002). When the plant is subjected to salt stress for a long time, the growth of the plant is slow, the membrane permeability is increased, and the photosynthesis is blocked (Wang et al., 2006; Zheng et al., 2010; Guo et al., 2013; Li and Zhang, 2013). In rapeseed, the seedling height, root length, aboveground fresh weight, dry weight, underground dry weight, and fresh weight are significantly reduced under salt stress (Wani et al., 2013). Salt stress also affects the physiological indexes of plants. Under salt stress, the content of K^+^ and Ca^2+^ in rapeseed seedlings decreases significantly, while the content of Na^+^ and Cl^−^ increases significantly, the growth of plants is inhibited, and Ca^2+^/Na^+^ and K^+^/Na^+^ decrease with the increase of salt concentration (Peng et al., 2004). When the plant is under salt stress, a large amount of active oxygen is generated, unbalancing the active oxygen metabolism and leading to changes in the activities of peroxidase (POD), catalase (CAT) and superoxide dismutase (SOD). These indexes in plant tissues are closely related to plant stress resistance (Zhang, 2012; Du et al., 2013; Wani et al., 2013). A similar phenomenon was also observed in chloroplasts, which is sensitive to salt stress. When the plants are subjected to salt stress, the chloroplast structure is destroyed, the chlorophyll content is reduced, photosynthesis is weakened and the growth is inhibited (Yang et al., 2010; Zhang, 2012). When salt stress is applied, plants also resist to salt damage by accumulating osmotic protection substances, such as betaine, proline, soluble sugar, and soluble protein, which are the main osmotic regulators in plants (Thakur and Sharma, 2005). Therefore, the growth characteristic of plants is a comprehensive response to salt stress (Kaya et al., 2002).

The complexity of most mechanisms involved in salt stress tolerance limits the development of modern salt tolerance breeding approaches (Yeo and Flowers, 1986). The association and application of the trait (s) of interest such as salt stress toleranceis a well-known approach for the betterment of crops (Im et al., 2014; Kumar et al., 2015). In order to study the mechanism of salt tolerance, researchers used the genetic, and molecular biology methods to map or clone the salt tolerance related genes. Currently, a few salt stress related QTLs detected by simple sequence repeat (SSR) markers have been identified, and these studies are mainly concentrated in wheat (Gorham et al., 1990; Dubcovsky et al., 1996; Lindsay et al., 2004; Huang et al., 2006), rice (Yao et al., 2005; Sabouri and Sabouri, 2008; Ahmadi and Fotokian, 2011) and so on. These QTLs are mainly focused on the Na^+^/K^+^ ratio in the root, the content of Na^+^, the root length, and root dry weight. In *Arabidopsis thaliana*, some QTLs and salt related genes have also been identified, such as *RAS1*, a gene encoding a plant-specific expression protein which is involved in plant salt tolerance at germination and seedling stages (Ren et al., 2010); *AtPirin1* was cloned in *Arabidopsis*, which can reduce the seed germination rate, and inhibit seedling growth under salt stress (Nguyen et al., 2007). There are also some studies on salt tolerance in the *Brassica* species, which were focused on the evaluation of salt tolerance (Jain et al., 1990; Su et al., 2013), salt tolerance homolog gene cloning and expression (Chakraborty et al., 2012). However, few studies on genetics and gene mapping of salt tolerance in *B. napus* were reported. *B. napus* is the main oilseed species in the world because of its considerable economic and nutritional value. The seed germination in *B. napus* is sensitive to salt stress and their growth and oil production are markedly reduced by salinity. Thus, it is imperative to study the salt tolerance in *B. napus*, cultivate the salt tolerance varieties, enlarge the planting area, so as to increase the amount of rapeseed and alleviate the edible oil gap.

In the previous study, many salt-tolerant breeding lines have been identified in our lab. For example, *B. napus* line 2205 derived from the restorer line (Z716C) of a *B. napus* cultivar “Shanyou0913,” was discovered through the salt treatment experiment (Huang et al., 2010), which shows a good correlation between salt tolerance and some morphological and physiological indexes. A further study shows this salt-tolerant line can still grow well under high salt concentration (Ding et al., 2014). Therefore, this line has been used as the source of salt tolerance in *B. napus*. The purpose of this study is to identify the QTLs related to salt tolerance, isolate the salt tolerance related genes in the QTL regions, and analyze the structure and expression of the candidate genes. In addition, hopefully to develop new salt tolerance related markers for marker assisted selection (MAS) of salt tolerance.

## Materials and methods

### Plant materials and stress treatments

An F_2_ population including 196 individuals derived from two *B. napus* lines, 2205 (salt-tolerant) and 1423 (salt-sensitive) was constructed for QTL mapping. Each individual of F_2_ was selfed to generate 196 F_2:3_ lines, which were used to measure morphological and physiological indexes. Ten plants of each F_2:3_ line were measured per replicate. A total of three replicates were assessed, first two replicates were conducted outdoors in the spring of 2013 and 2014, respectively (15–25°C), and the third one was cultivated in a light incubator in 2015 (16/8 h, 25/20°C). Hydroponic growth was used to cultivate the two parents, F_1_ and F_2:3_ populations, in brief, the seeds of them were first disinfected with 70% alcohol, soaked in distilled water for 12 h, and then placed in a glass dish to germinate for 48 h under dark conditions. When the buds were 5–7 cm, the plants were transferred to l/2 Hoagland nutrient solution (pH 6.0), until three leaves appeared, the plants were treated with 200 mM NaCl for 10 days. In addition, the two parents 2,205 and 1423 were also treated with 0, 100, and 200 mM NaCl at three leaves time, respectively. The roots and leaves of the two parents were sampled at 0, 6, 12, and 24 h, respectively. The samples were stored at −80°C for RNA extraction.

### Evaluation of morphological and physiological indexes

After 10 days of the salt treatment, 10 morphological and physiological indexes were determined, including root length (RL), leaf fresh weight (LFW), leaf dry weight (LDW), root dry weight (RDW), electrical conductivity (EC), superoxide dismutase (SOD), soluble protein (SP), chlorophyll content (SPAD), salt tolerance rating (STR), and seedling height (SH). A total of 10 individuals from each F_2:3_ line were measured, and the average was calculated as the measured value of each index. EC was determined by the method of Li (2000); SOD was measured by the method of Beauchamp and Fridovich (1971); SP was determined by the Coomassie Brilliant Blue method described by Li (2000) and the chlorophyll content was measured on a SPAD-502 plus (Konica Minolta, Japan) following the manufacturer's instructions.

### Extraction of DNA, SSR, and amplified fragment length polymorphism (AFLP) technology

Genomic DNA was extracted from the young leaves of F_2_ individuals at the seedling stage by the cetyl trimethyl ammonium bromide (CTAB) method (Doyle and Doyle, 1990). The final DNA concentration was adjusted to 50 ng/μl. Sequences of all SSR markers were obtained from public sources: including the Databases on http://ukcrop.net/perl/ace/search/BrassicaDB (Lowe et al., 2004) and http://www.brassica.info/resource/markers.php (for those with the prefixes: Ra, Na, BN, and BRMS-), the electronic supplementary material of Piquemal et al. (2005) (for those primer pairs with the prefixes “BRAS” and “CB”), and the other ones were from the studies of Cheng et al. (2009) and Xu et al. (2010) (for those with the prefixes “Bra,” “Bro,” and “Brn”). The PCR products were detected by silver staining (Lu et al., 2001). The AFLP method was described by Vos et al. (1995), and the sequences of the pre-amplified primers and selective amplified primers were from Huang et al. (2007).

### Genetic linkage map construction and QTL analysis

An AFLP and SSR linkage map was established using the MAPMAKER 3.0 program (Lander et al., 1987; Lincoln et al., 1992). A minimum log likelihood of the odds (LOD) score of 3.0 and a maximum distance of 25 cM were used to group loci into linkage groups (LGs). Map distances were calculated using the Kosambi's mapping function (1944). The QTLs were mapped using the composite interval mapping (CIM) function of the Win QTL Cartographer v.2.5 (Basten et al., 1997). The LOD thresholds of QTL were determined by a 1,000 permutation test at a 95% confidence level. The QTL mapping was performed followed the method of (Fan et al., 2010).

### Fine mapping of the candidate genes

The QTLs detected in all three replicates were selected. The products of the two closest markers around the QTL were sequenced. The collinearity between the sequences of the two markers and the A genome was compared using the BLAST tool (http://brassicadb.org/brad). After the physical position of the QTL on the A genome was determined, the genes within the QTL region were randomly selected for designing intron polymorphism (IP) primers. The detailed method can be referred to Huang et al. (2016). All IP primers were used to amplify the parents and F_2_ population. WinQTLcart2.5 was used to analyze phenotypes and polymorphic bands.

### Total RNA extraction and cDNA synthesis

A RNA prep Pure Plant Kit (TIAN GEN, Beijing, China) was used for total RNA extraction from the root and leaf tissues of the two parents' seedlings that were ground in liquid nitrogen after being processed for 0, 6, 12, and 24 h with 0, 100, and 200 mM NaCl. After DNA removal by DNaseI digestion, agarose gel (mass-to-volume ratio was 1.2%) electrophoresis was performed to measure RNA concentration and integrity. cDNA synthesis was performed following the instructions of the TIAN GEN FastQuant RT Kit (Beijing, China). These cDNAs were used for analyses of candidate gene cloning and expression.

### Amplification of the candidate genes

After the candidate genes were selected in the QTL region, the RT-qPCR technique was applied to amplify the full-length ORF of the cDNA from the leaf tissues of the two parents' seedlings. The primer sequences were: Forward, 5′-ATGAAGATTCAGTGTAACGTCTGCGAGA-3′; Reverse, 5′-TTAGAACCGTCTTCGCTTCCCAACCCTCT-3′. The full-length DNA sequence was amplified using the genomic DNA as a template, and the amplification reaction was as follows: denaturation at 95°C for 10 s, followed by renaturation at 55°C for 5 s, and elongation at 72°C for 1 min for a total of 35 cycles. The Prime STAR Taq DNA polymerase (Takara) was added once the PCR program was suspended. PCR products were purified by a TIAN gel Midi Purification Kit (TIAN GEN, Beijing, China). The purified products were connected to the carrier of the pMD19-T vector, and transformed to *Escherichia coli* DH5α cells. A total of 10 positive clones were sent to Shanghai Sangon Biological Engineering Technology & Service Co., Ltd for sequencing. Full-length cDNA sequences were analyzed by the DNAMAN5.0 software. Multiple sequence alignment of the amino acid sequences was performed using the Clustal W. Phylogenetic tree was constructed by the ML (maximum likelihood) method with bootstrap analysis (1,000 replicates) from alignment of protein sequences of candidate genes in *Arabidopsis, B. rapa, B. napus*, and *B. oleracea* using MEGA5.0 program. The conserved domains, homologs and physicochemical properties of amino acids were analyzed by SMART (http://smart.embl-heidelberg.de/), Blast (http://blast.ncbi.nlm.nih.gov/Blast.cgi) and PROTPARAM (http://web.expasy.org/cgi-bin/protparam), respectively.

### Expression of the candidate genes

RT-qPCR was used to analyze the expression of the candidate genes in the root tissues and leaf tissues from the two parents' seedlings. The SYBR Green Supermix kit purchased from Takara was used for PCR reactions, and the reaction conditions were as follows: pre-denaturation at 95°C for 2.5 min, followed by denaturation at 95°C for 10 s, and renaturation at 60°C for 34 s for a total of 40 cycles; at the end of the reaction, the system was kept at 95°C for 15 s, followed by lowering the temperature to 60°C for 1 min. The replicates were carried out for each experiment following the manufacturer's instructions. The forward primer sequence was 5′-GAAGCATTTCTCAGTGGCAA-3′, and the reverse primer sequence was 5′-ATGATGTCTCACCTCCTCCC-3′. β-actin was used as an internal reference, with a forward primer sequence of 5′-TCAAGAAGGCTATCAAGGAG-3′ and the reverse primer sequence of 5′-GTAACCCCATTCGTTGTCAT-3′.

## Results

### Phenotypic variation

**Figure 1:**
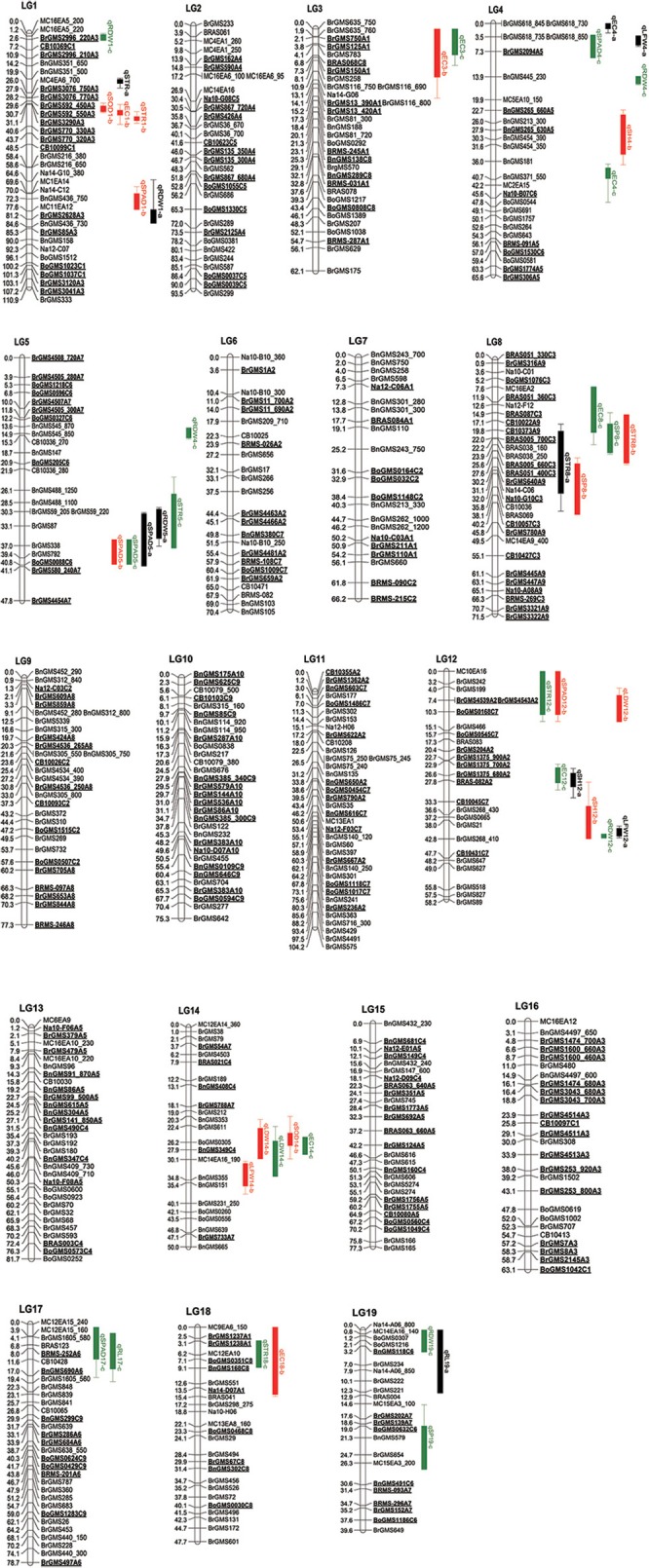
Genetic map of *B. napus* with the locations of putative QTLs for salt tolerance based on F_2:3_ populations. The numbers of linkage groups are shown at the top, the genetic distance (cM) and markers are indicated at the left and right, respectively. The QTLs for ten indexes are signed on the right of linkage groups.

The phenotypic distribution of traits related to salt tolerance in the two parents and 196 F_2:3_ families are shown in Figure 1. There were differences in all traits between the two parents under 200 mM NaCl (Table 1). STR and EC of the salt-resistant parent 2205 were higher than those of the salt-sensitive parent 1423, whereas the other traits of 2205 were lower than those of 1423. There were continuous frequency distribution and transgressive segregation in these indicators among F_2:3_ populations under salt stress (Figure 1). The normality test of the observed data in the F_2:3_ lines showed that the skewness and kurtosis were not significant (Table 1), indicating a normal distribution of all studied traits.

**Table 1:** Phenotypic performance of salt-tolerance traits in two parents and F_2:3_ population under 200 mM NaCl.

**Traits^a^**	**Parents^b^**		**F**_**2:3**_ **Population^c^**					
	**2205**	**1423**	**Mean**	**Min**	**Max**	**SD**	**Skewness**	**Kurtosis**
STR	0	3	0.74	0.00	2.00	0.429	0.505	0.338
SPAD	31.80	24.60	33.05	27.50	39.07	2.895	0.025	−0.307
EC	0.48	0.95	0.69	0.49	0.91	0.13	0.047	−0.631
RL	7.50	6.30	6.47	5.02	8.80	2.021	0.452	−0.193
SH	10.30	7.20	18.62	13.95	22.95	1.405	−0.018	−0.549
SP	17.90	5.65	9.26	2.87	17.94	3.240	0.001	−0.352
SOD	30.00	15.00	26.70	13.95	45.72	6.870	0.345	−0.192
LDW	0.04	0.01	0.02	0.01	0.03	0.014	0.268	0.388
RDW	0.05	0.02	0.05	0.02	0.09	0.015	0.499	−0.137
LFW	0.47	0.31	0.21	0.10	0.37	0.142	0.791	0.890

a *STR, salt tolerance rank; SPAD, chlorophyll; EC, electric conductivity; RL, root length; SH, seed height; SP, solution protein; SOD, superoxide dismutase; LDW, leaf dry weight; RDW, root dry weight; LFW, leaf fresh weight*.

b *Parents repeated in each sample measuring three times and presenting the mean value*.

c *F_2:3_ population size n = 196, replicates r = 3*.

### Correlation of all indicators

As shown in Table 2, there were significantly negative correlations among STR, SPAD, and RDW; a significantly positive correlation was found between STR and EC. SPAD was also significantly positively correlated with SP. EC was significantly negatively correlated with RL, RDW, and SFW. RL was significantly negatively correlated with SDW and SFW. SH was only significantly positively correlated with SDW. SP was significantly correlated with SPAD. SOD was only significantly negatively correlated with RDW. SDW was also significantly positively correlated with RDW and SFW. RDW was significantly positively or negatively correlated with STR, EC, SOD, and SDW.

**Table 2:** Correlation coefficient between different indexes under 200 mM NaCl.

	**SPAD**	**EC**	**RL**	**SH**	**SP**	**SOD**	**SDW**	**RDW**	**SFW**
STR	−0.213^**^	0.212^**^	−0.062	0.044	0.08	0.178	−0.029	−0.213^*^	−0.196
SPAD		−0.329^*^	−0.026	−0.041	0.219^*^	−0.149	0.199	0.125	0.068
EC			−0.279^*^	−0.107	−0.089	0.028	−0.309^*^	−0.276^*^	−0.215^*^
RL				0.041	0.028	−0.183	0.598^*^	0.107	0.674^*^
SH					−0.106	0.005	0.302^*^	0.170	0.170
SP						−0.11	−0.099	−0.179	−0.153
SOD							−0.059	−0.231^*^	−0.092
SDW								0.213^*^	0.815^*^
RDW									0.108

** and * *Indicates significance at the level of 1 and 5%, respectively*.

### Construction of the genetic linkage map

A total of 1,800 pairs of SSR and 64 pairs of AFLP primers were used to screen the two parents, 2205 and 1423. Among these, 426 pairs of SSR and 15 pairs of AFLP primers showed polymorphism between the two parental lines, with 24 and 23%, respectively. These polymorphic primers were then used to screen the F_2_ population, from which 531SSR polymorphic and 30 AFLP loci were identified. And a total of 502 SSR and 30 AFLP markers were grouped into 19 linkage groups with an LOD threshold of 3.0. The map spanned 1341.1 cM with an average marker interval of 2.52 cM. The markers were distributed relatively evenly, with the longest linkage group (LG1) containing 34 markers spanning 110.85 cM and the shortest (LG19) containing 23 markers spanning 39.6 cM. The LGs corresponded to the 19 chromosomes of *B. napus* including A1–A10 (A genome) and C1–C9 (C genome) as determined by shared SSR markers in public genetic maps (Cheng et al., 2009; Xu et al., 2010). A total of 131 SSR markers of the map were from the A genome, and 70 SSR markers were from the C genome. Because the A and C genomes share homology, partial linkage groups contain both the A and C genomic molecular markers, such as LG2, LG8, LG10, and so on (Table 3).

**Table 3:** Detailed information of genetic map constructed in the F2 population.

**Linkage groups**	**Map length (cM)**	**No. of loci**	**No. of SSR on A genome**	**No. of SSR C genome**	**Map density (cM/marker)**
1	110.85	34	A3/13	C1/4	3.26
2	93.5	31	A4/8	C5/6	3.01
3	62.1	32	A1/8	C8/4	1.94
4	65.6	26	A5/6	C6/2	2.52
5	47.8	23	A7/7	C6/5	2.08
6	70.4	24	A2/8	C7/3	2.93
7	66.2	22	A1/5	C2/5	3.01
8	71.5	30	A9/10	C3/11	2.38
9	77.3	30	A8/10	C2/5	2.58
10	75.3	31	A10/9	C9/8	2.43
11	104.2	37	A2/5	C7/2	2.82
12	58.2	25	A2/5	C7/2	2.33
13	81.7	32	A5/9	C4/2	2.55
14	50.0	23	A7/1	C4/1	2.17
15	77.3	27	A5/6	C4/3	2.86
16	63.1	26	A3/11	C1/1	2.42
17	78.7	31	A6/4	C9/1	2.54
18	47.7	25	A1/3	C8/3	1.91
19	39.6	23	A7/3	C6/2	1.72
Total/mean	1341.1	532	131	70	2.52

### QTL mapping of salt tolerance at the seedling stage

Forty-five QTLs controlling 10 traits were mapped on the linkage map (Table 4, Figure 1). Among them, seven QTLs associated with STR were detected on LG1, LG5, LG8, LG12, and LG18, respectively. Two QTLs (qSTR1-a and qSTR1-b) were detected in the same loci of LG1, and two QTLs (qSTR8-a and qSTR8-b) were identified in the same loci of LG8. All QTLs related to STR can explain more than 10% of the total phenotypic variance, and the QTL (qSTR18-c) on the LG18 accounted for 36.35% of the total phenotypic variance. For SPAD, seven QTLs were mapped to five LGs. Three QTLs (qSPAD5-a, qSPAD5-b and qSPAD5 c) were located on the same loci of LG5 and showed relatively large effects on SPAD, accounting for 25.11, 37.49, and 51.14% of the total phenotypic variation, respectively. These QTLs had positive additive effects for increased SPAD from 2205. The other QTLs related to SPAD were detected on LG1, LG4, LG12, and LG17. For EC, seven QTLs were detected on LG1, LG3, LG4, LG8, LG12, LG14, and LG18. Among them, two QTLs (qEC3-a and qEC3-b) were detected in the same region of LG3. qEC12-c located on the LG12 had the highest *r*^2^ among all QTLs, accounting for 21.37% of the total phenotypic variation. All QTLs had positive additive effect for the increased EC. Five QTLs were identified for RDW, among which, qRDW1-a and qRDW4-c showed the largest effect on RDW, accounting for 23.56 and 18.9% of the total phenotypic variance, respectively. Only two QTLs (qSOD1-b and qSOD14-b) for SOD were mapped to LG1 and LG14, respectively. They accounted for 19.7 and 27.5% of the total phenotypic variance and showed positive additive effect for the increased SOD. For SP, three QTLs were detected on LG8 (qSP8-b and qSP8-c) and LG19 (qSP19-c), which accounted for 12.91, 8.9, and 16.7% of the total phenotypic variance respectively. Three QTLs for SH were identified on LG4 and LG12, among which, qSH12-a and qSH12-b located on the LG12 were adjacent to each other. qSH4-b and qSH12-a, had the highest *r*^2^ and accounted for 22.3 and 26.9% of the total phenotypic variance respectively. These two QTLs showed negative additive effect for the decreased SH. For RL, two QTLs (qRL17-c and qRL19-a) were detected on LG17 and LG19, which accounted for 16.2 and 8.7% of the total phenotypic variance, respectively. These two QTLs showed positive additive effect for the increased RL. For LFW, three QTLs were mapped to LG4 (qLFW4-a), LG12 (qLFW12-a), and LG14 (qLFW14-b), which accounted for 24.3, 29.9, and 16.54% of the total phenotypic variance, respectively. Two (qLF4-a and qLF14-b) showed positive additive effects for the increased LF and one (qLF12-a) showed negative additive effect for the decreased LF. Three QTLs for LDW were mapped to LG12 (qLDW12-b) and LG14 (qLDW14-b and qLDW14-c) respectively, which accounted for 12.1, 14.2, and 20.74% of the total phenotypic variance, respectively. By comparing the QTLs of different indexes, it was found that many QTLs from different indexes were mapped to the same region of the linkage group (Figure 1). The QTLs, qSTR1-a, qSTR1-b, qSOD1-b, and qEC1-b, were mapped to the same region of LG1. qSPAD1-b and qRDW1-a were detected in another region of LG1. Three QTLs, qSPAD4-c, qEC4-a, and qLFW4-a were mapped to the same area of LG4. Three QTLs (qSPAD5-a, qSPAD5-b, and qSPAD5-c) for SPAD, one QTL for STR (q STR5-c) and one QTL for RDW (qRDW5-a) were detected in the same region of LG5. Two QTLs (qSTR8-a and qSTR8-b) for STR, two QTLs (qSP8-b and qSP8-c) for SP and one QTL (qEC8-c) for EC were also clustered to the similar area of LG8. Five QTLs for four indexes were also identified in the same area of LG14 (Figure 1).

**Table 4:** Detailed information of QTLs for relevant indexes.

**Traits^a^**	**LGs^b^**	**QTLs**	**Intervals**	**LOD**	***r*^2^ (%)^c^**	**Add^d^**	**D^e^**
STR	1	qSTR1-a	MC4EA6_700-BrGMS3076_750	3.7	14.43	0.26	0.11
	1	qSTR1-b	BrGMS592_550-BrGMS3290	4.2	17.3	0.2	−0.08
	5	qSTR5-c	BnGMS488_1100- BrGMS792	2.8	12.61	0.21	−0.03
	8	qSTR8-a	BRAS005_700-BRAS069	2.9	17.44	0.12	0.12
	8	qSTR8-b	CB10022- BRAS005_700	2.6	13.14	−0.15	0.58
	12	qSTR12-c	MC10EA16-BoGMS0168	2.9	10.42	−0.21	−0.3
	18	qSTR18-c	BrGMS1238-BnGMS168	3.1	36.35	0.4	−0.48
SPAD	1	qSPAD1-b	Na14-C12-MC11EA12	3	32.45	−0.78	−0.21
	4	qSPAD4-c	BrGMS618_735- BrGMS2094	2.5	21.18	−1.27	0.63
	5	qSPAD5-b	BrGMS338-BrGMS580_240	4.8	25.11	0.64	0.15
	5	qSPAD5-c	BrGMS338- BrGMS580_240	6	37.49	0.83	0.27
	5	qSPAD5-a	BrGMS59_205-BrGMS580_240	4.8	51.14	1.13	1.8
	12	qSPAD12-b	MC10EA16- BoGMS0168	3.1	18.1	−2.02	0.64
	17	qSPAD17-c	MC12EA15_160-CB10428	3.1	19.7	1.58	0.24
EC	1	qEC1-b	BrGMS592_450- BrGMS592_550	3	15.3	0.37	−0.5
	3	qEC3-a	BrGMS635_760- BrGMS783	6.7	7.5	1.71	0.63
	3	qEC3-b	BrGMS635_760- BrGMS150	7.1	11.83	0.79	0.75
	4	qEC4-a	BrGMS618_845- BrGMS618_735	3	4.8	0.3	−0.19
	4	qEC4-c	BnGMS181- BnGMS371_550	3.5	11.5	0.91	0.27
	8	qEC8-c	MC16EA2-CB10373	2.8	12.9	0.74	0.63
	12	qEC12-c	BrGMS1375_900- BrGMS1375_700	3.2	21.37	0.45	−0.45
	14	qEC14-c	BrGMS611-BnGMS349	3.2	6.3	1.36	0.42
	18	qEC18-b	MC9EA6_150-BRAS041	2.5	4.1	1.61	0.63
RDW	1	qRDW1-c	MC16EA_220-BrGMS2996_220	2.6	5.27	−0.99	0.77
	1	qRDW1-a	MC11EA12-BnGMS436_735	2.4	23.56	1.09	0.28
	4	qRDW4-c	BnGMS445_230-MC5EA10_150	11.9	18.9	0.71	0.27
	5	qRDW5-a	BrGMS59_205- BrGMS171	15	10.48	−1.45	0.38
	12	qRDW12-c	BrGMS21- BrGMS268_410	20.7	51.8	0.87	0.75
	19	qRDW19-c	MC14EA16_140-BnGMS118	3.2	9.8	1.39	−0.18
SOD	1	qSOD1-b	BrGMS592_450- BrGMS592_550	3.2	19.7	1.5	−0.14
	14	qSOD14-b	BrGMS611- BnGMS349	3.8	27.5	2.2	0.71
SP	8	qSP8-b	BRAS005_660-BRAS069	4.5	12.91	−1.03	0.28
	8	qSP8-c	CB10022- BRAS038_160	5.5	8.9	0.3	0.23
	19	qSP19-c	BoGMS0632-MC15EA3_200	3.6	16.7	0.13	0.17
SH	4	qSH4-b	BnGMS213_300- BnGMS181	3.7	22.3	−0.2	−1.31
	12	qSH12-a	BrGMS1375_700- CB10045	4.5	26.9	−0.45	0.83
	12	qSH12-b	BrGMS268_430- BrGMS268_410	4.1	6.12	1.07	−0.89
RL	17	qRL17-c	MC12EA15_160-BnGMS690	4.8	16.2	1.44	2.01
	19	qRL19-a	MC14EA16_140-BrGMS211	3.7	8.7	0.98	0.14
LFW	4	qLFW4-a	BrGMS618_735- BrGMS2094	3.9	24.3	0.37	0.15
	12	qLFW12-a	BrGMS21- BrGMS268_410	3.8	29.9	−0.68	0.43
	14	qLFW14-b	MC14EA16_190- BnGMS151	4.5	16.54	0.84	0.35
LDW	12	qLDW12-b	BrGMS4539- BrGMS466	5.5	12.1	0.22	0.11
	14	qLDW14-b	BrGMS611-MC14EA16_190	3.7	14.2	0.36	0.45
	14	qLDW14-c	BoGMS0305- BnGMS355	3.5	20.74	−0.21	−0.21

a Traits: ten salt tolerance indicators;

b LGs: linkage groups on which the QTL was located;

c r^2^: Variation accounted for by each putative QTL;

d *Add: Additive effect*.

e *D: Dominant effect*.

### Fine mapping of the salt tolerance gene on LG5

**Figure 2:**
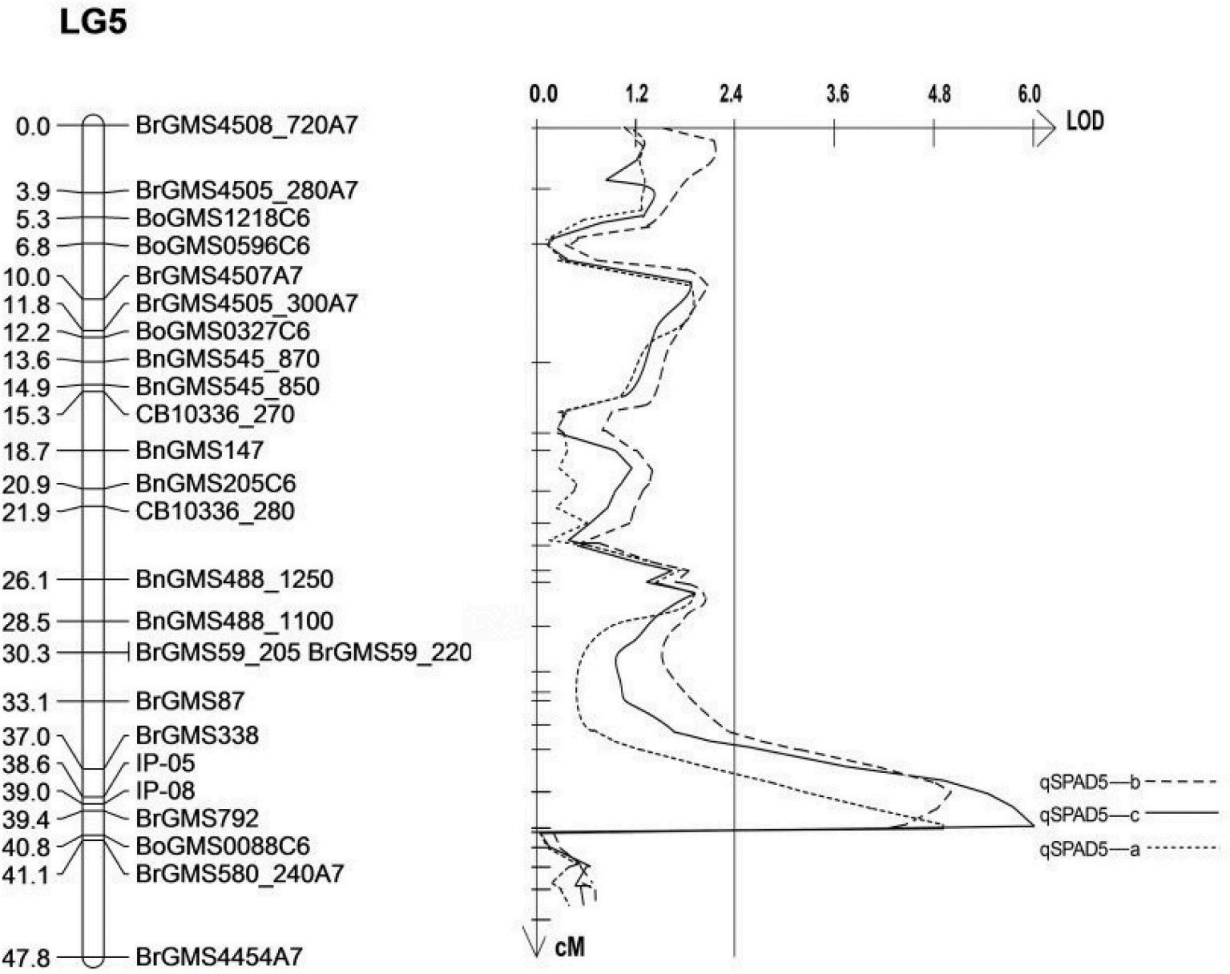
Mapping of QTLs related to salt tolerance on LG5. The markers names are shown to the right of the linkage group (LG5), and genetic distance (cM) is on the left. The vertical line is LOD threshold for QTL.

As three QTLs related to SPAD on the LG5 were detected in the same area, it was speculated that they are the same loci, which means the same QTL can be detected in three replicates. Therefore, the sequences of the markers on either sides of the QTL (BrGMS338, BrGMS792 and BrGMS580_240) were analyzed. The result showed that the QTL was located between 16.33 and 23.14 Mb on A07 of *B. rapa*. In order to narrow down the region of the QTL, 20 gene sequences within this region were selected to design IP primers. Two IP primers, IP05 and IP08 were successfully designed to amplify the polymorphic bands between the two parents and F_2_populations. Win QTL Cartographer v.2.5 was used to analyze the phenotypes and polymorphic bands. The interval of the QTL was finally mapped to a interval between IP05 (17.41 Mb) and BrGMS792 (17.80 Mb) with a region of 390 Kb (Table 5, Figure 2). A total of 66 genes were identified in this region by comparing the database (http://brassicadb.org/brad/). By analyzing the functional annotation of each gene in this region, one gene, *Bra003640*, was found to be associated with salt tolerance in plants. Thus, *Bra003640* was further investigated as a candidate gene for this study.

**Table 5:** Information of molecular markers linked to qSPAD5.

**Markers**	**Sequences (5′–3′)**	**Location (Mb)**
BrGMS338	CTAGGGAGAGAGAACAACTCGC/GTGCTCAAAGATACACGTTTCG	16.337
BrGMS792	CATTGTCCCCATCTCTTACCAT/GAAGGAGGTGAGTTTGAGCTTG	17.874
BrGMS580_240	ATATCGAGCGGTACGGAGATTA/CGATTTCTAGGGTTCTTCCTCC	23.140
IP05	TAACACTTGCATTCGCGGTG/GGTCCATAGTCACCATCTCCG	17.410
IP08	CCTCTGCTTTCTTTCGCATCA/GGCGTGAGTCAGAGGAAGAC	17.602

### Structural analysis of the salt tolerant candidate gene

**Figure 3:**
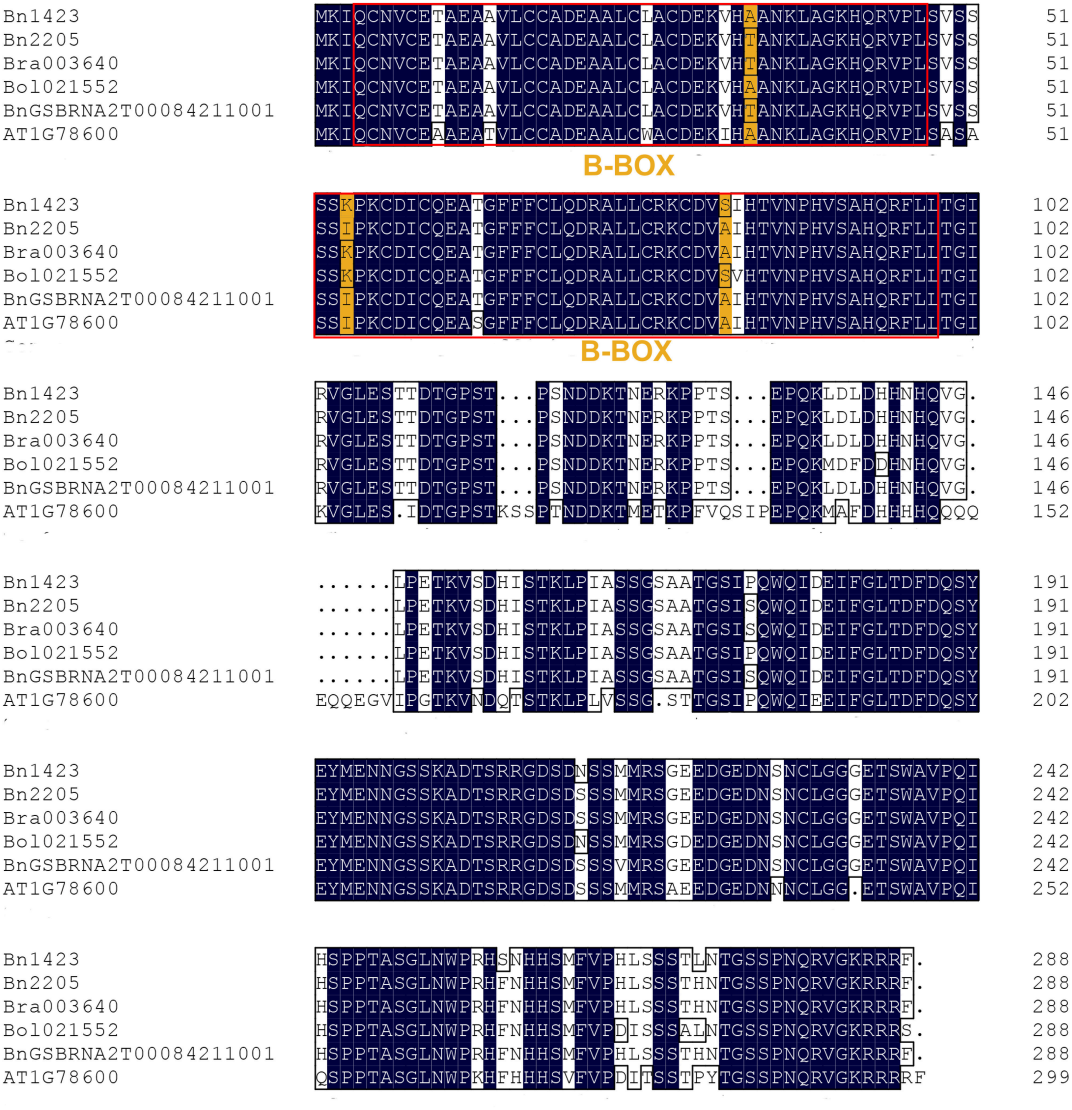
Sequences comparison of *Bra003640* between *Arabidopsis* and *Brassica* species. The red rectangle indicates the two B-box zinc finger protein domains. The mutant amino acids between the two parents in B-box zinc finger protein domains are indicated in yellow, the other mutant amino acids are indicated in white.

The candidate gene was amplified in the two parents 2205 and 1423 by homologous cloning according to the sequence of *Bra003640*. These two genes were named as *Bn2205* and *Bn1423*, respectively. The full-length of the genes in both 2205 and 1423 were 1,063 bp, which contained three exons and two introns. The ORFs were 867 bp encoding a protein of 287 amino acids, with the isoelectric points of 5.99 and 6.04, and the molecular weights of 31.34 and 31.30 kDa, respectively. The ORF sequences of *Bn2205* and *Bn1423* were compared by the DNAMAN5.0 software. The results showed there were 11 single nucleotide differences between them, resulting in seven amino acid changes (34, 54, 83, 174, 212, 257, and 272) (Table 6, Figure 3). Two typical B-box zinc finger protein domains were found between residues 4–46 and 52–99, respectively, by comparing the conserved domain database (Figure 3). Among them, the amino acids 57–90 are typical C2C2 and C2H2 type zinc fingers. Three amino acid changes (34, 54, and 83) were identified in the conserved domain between the two parents. The amino acid sequences of the two genes were aligned to those from the *Arabidopsis thaliana* database (http://www.Arabidopsis.Org/), which showed the closest homologous was *Arabidopsis* BBX22.

**Table 6:** Structure analysis of Bn2205 and Bn1423.

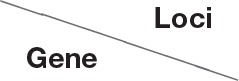	**34**	**54**	**83**	**174**	**212**	**257**	**272**
*Bn2205* (Amino acids)	T	I	A	S	S	F	H
*Bn1423* (Amino acids)	A	K	S	P	N	S	L

**Figure 4:**
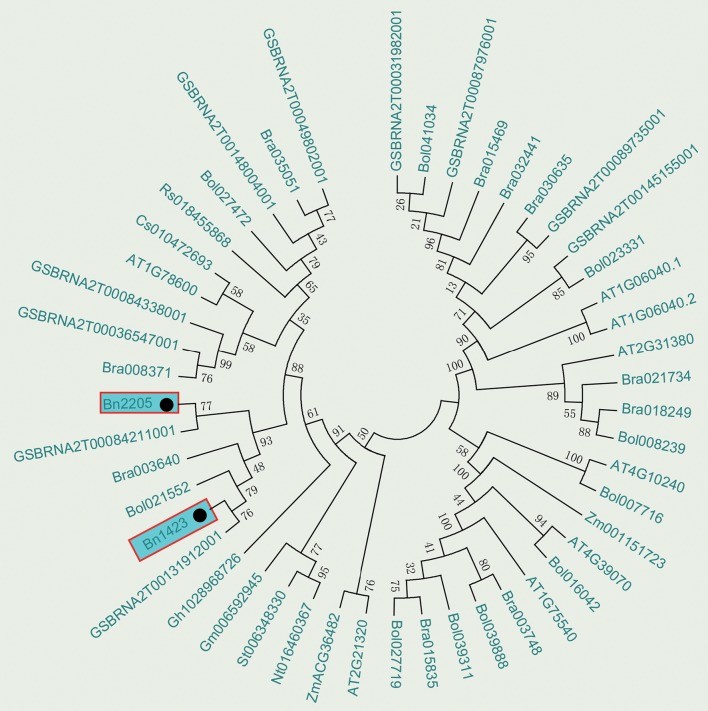
Phylogenetic tree analysis of the candidate genes. Numbers are bootstrap values indicating frequencies of respective furcations found in 1,000 replications of subset tree calculations.

The BLAST tool developed by NCBI was used to search for the B-box zinc finger proteins in *Brassica* species reported in GenBank and the phylogenetic analysis was conducted. The results showed that *Bra003640* from *B. rapa, Bol021552* from *B. oleracea*, and *GSBRNA2T00084211001* and *GSBRNA2T00131912001* from *B. napus* were clustered together with *Bn2205* and *Bn1423* (Figure 4). All above genes had the function of salt stress induction. Therefore, it is presumed that the candidate gene can respond to salt stress. A sequence of 1,500 bp upstream of the ATG of *Bn2205* and *Bn1423* was analyzed for cis-acting elements using Plant CARE. Table 7 showed that a number of cis-acting elements AF1, HD-Zip 1, LTR, and HSE are related to plant stress tolerance and transcriptional activity, suggesting that the *Bra003640* gene may be induced by abiotic stresses.

**Table 7:** Analysis of cis-acting elements in the upstream of the candidate gene.

**Cis-acting elements**	**Core sequence**	**Annotation**
3-AF1 binding site	AAGAGATATTT	Light responsive element
Box I	TTTCAAA	Light responsive element
CAT-box	GCCACT	Meristem expressionelement
HD-Zip 1	CAAT(A/T)ATTG	Palisade mesophyll cells
HD-Zip 2	CAAT(G/C)ATTG	Control of leaf morphology development
HSE	AAAAAATTTC	Heat stress responsivenesselement
LTR	CCGAAA	Low-temperature responsivenesselement

### Expression of the salt tolerant candidate gene

**Figure 5:**
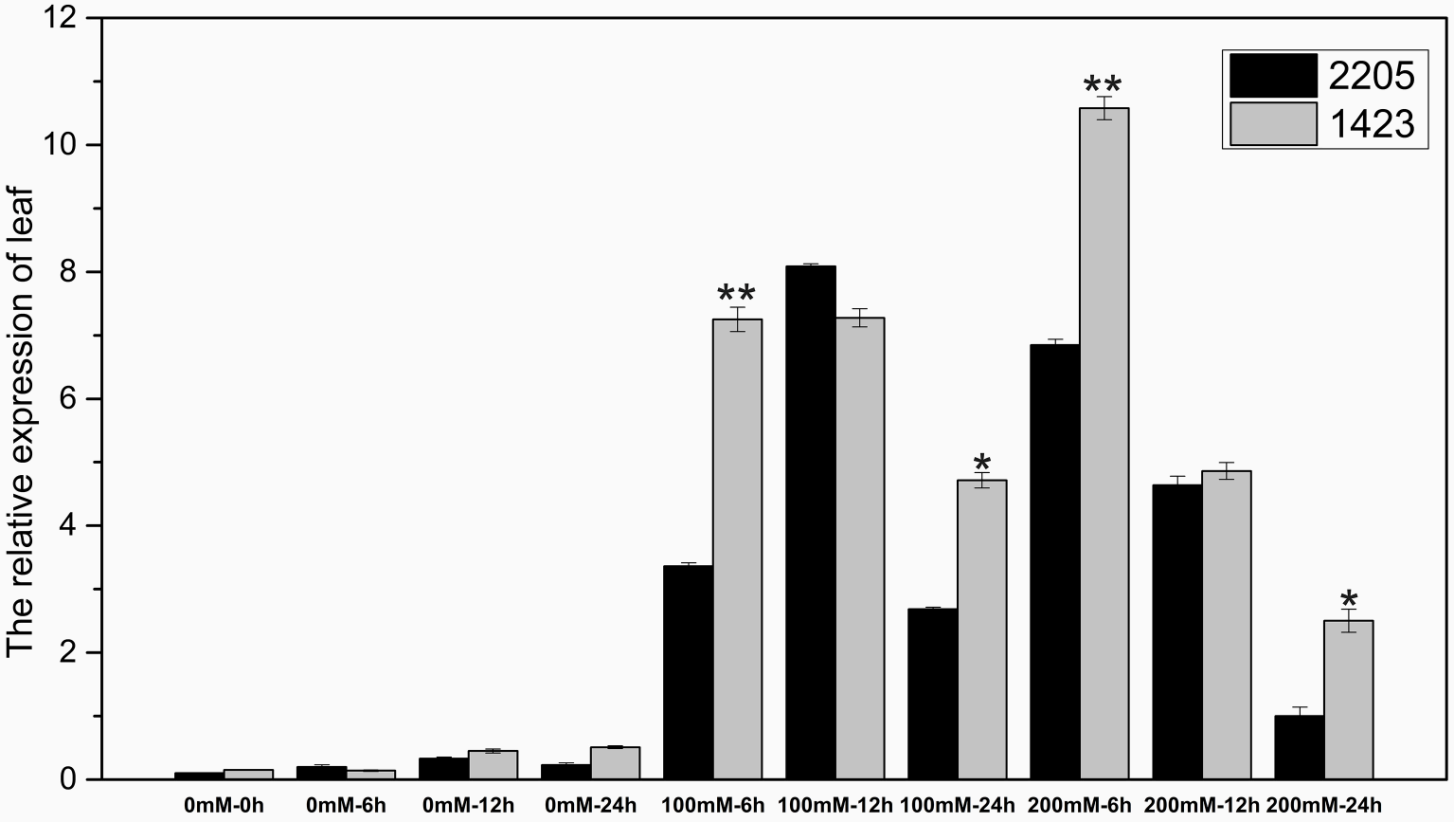
Expression of the candidate gene in leaves of the two parents. ^*^ and ^**^ stand for significance level at *P* < 0.05 and *P* < 0.01, respectively.

**Figure 6:**
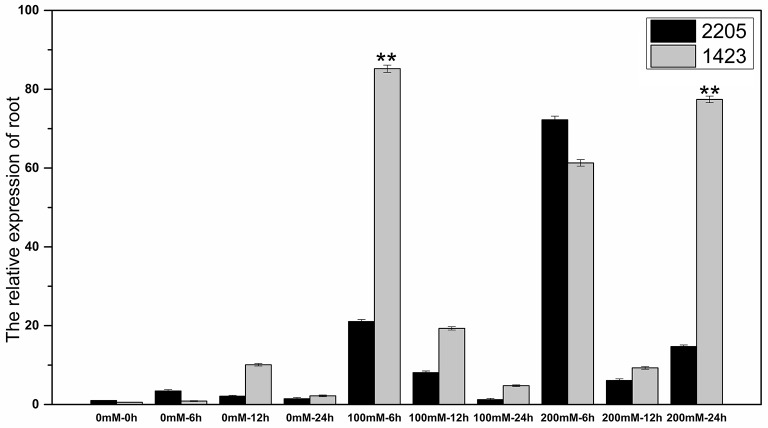
Expression of the candidate gene in roots of the two parents. ^**^ stands for significance level at *P* < 0.01.

The RT-qPCR technique was used to analyze the expression of the candidate gene under the high salt treatment. The results showed that the expression of this gene was low without salt stress, but increased under high salt stress between the two parents. We had observed differential expression of the candidate gene in leaf and root samples between parents. In the leaves, the candidate gene was significant highly expressed in 1423 compared to 2205 at 6 h under 100 and 200 mM NaCl treatment (Figure 5). In the roots, comparing with 2205, the candidate gene was significant highly expressed in 1423 at 6 h under 100 mM whereas 24 h under 200 mM NaCl. The expression in both parents showed a downward trend with time, however, a drastic increase in expression was observed in 1423 at the 24 h under 200 mM NaCl (Figure 6). Therefore, our results show that the candidate gene is associated with salt stress.

## Discussion

Salinization of land is one of the crises the world is facing today; therefore, it is urgent to plant crops on the saline-alkali soil to meet human needs. At present, studies of salt tolerance are mainly focused on rice, wheat, cucumber and other crops (Lindsay et al., 2004; Huang et al., 2006; Wang et al., 2012; Ghomi et al., 2013; Song et al., 2016). However, little has been done on salt tolerance of rapeseed. Salt resistance of crops is complex due to interacting factors, some of which are only activated in salt stress. Thus, it is difficult to evaluate these factors under normal growth conditions. An effective way to evaluate salt resistance is by using the physiological and morphological indexes of plants under salt stress. In this study, a number of physiological and morphological indicators were used to determine the salt tolerance of *B. napus*, and some major QTLs related to salt tolerance were obtained. Some of them accounted for more than 20% of the phenotypic variance, such as qSPAD5-a, qSPAD5-b, qSPAD5-c, qSTR18-c and so on. Many QTLs for different indicators were mapped to the same region, such as the QTLs on LG1, LG4, LG5, LG8, and LG14. Our results suggest the salt-related QTLs we mapped are accurate and the presence of a single gene in this region controls multiple traits. Multiple indicators were used to analyze salt tolerance. Many salt tolerance-related QTLs were mapped to the same region, which provides a very reliable reference for the selection of salt-tolerant candidates in further studies.

Currently, only a few reports about the genetics of salt tolerance in rapeseed were published, and not too many genes have been mapped or cloned. Map-based cloning has long been considered as a reliable and effective means of gene cloning, and many genes have been cloned in *Brassica* using this method (Dun et al., 2011; Li et al., 2012). In this study, the map-based cloning technology was used to map and clone the salt tolerance related genes. A key step is the identification of candidate genes for salt tolerance, and this work is premised on fine mapping of salt-tolerant genes. Therefore, The QTL technology was used to identify the QTLs related with salt tolerance. The salt tolerance of rapeseed was indirectly measured by several indexes. Our results show a number of major QTLs related with salt tolerance were detected, and some of the QTLs were enriched in a specific region of the linkage group. Thus, these areas were identified as the candidate regions of salt-tolerant related genes. In this study, the QTL associated with SPAD on LG5 could be detected repeatedly in the same region, and several QTLs for other traits were also mapped to this region, therefore, there is a high probability of salt tolerance genes located in this region. However, the QTL region (qSPAD5-a, qSPAD5-b, qSPAD5-c) has 6.8 Mb in the initial QTL mapping, and there are hundreds of genes in this region, it is difficult to screen the genes related to salt tolerance. Fortunately, the sequence of the Chinese cabbage genome has been published, and some genes have very detailed functional annotations, which facilitate us to exploit the sequence of the genes in the QTL region to develop closer molecular markers. Two salt-related IP markers were developed to narrow down the QTL region to 390 kb. In fact, a similar approach has been used to fine map the target genes in previous studies (Huang et al., 2016). A total of 66 genes were identified in this region by analyzing the functional annotations of these genes. *Bra003640*, associated with salt tolerance, was identified for subsequent studies. Through gene structure and expression analysis, it was found that the gene sequence between the two parents was different, particularly the presence of three different amino acids within the conserved domain. We further verified its function by the transgenic technology. Additionally, for LG1, LG4 and other QTL enrichment intervals, a similar method was used to develop IP markers to narrow down the QTL interval. Meanwhile, in order to finely map the genes related to salt tolerance in these QTLs regions, an F_2:3_ population of 1,200 lines was constructed, and the phenotypic identification is ongoing in our lab.

As the salt tolerance of rapeseed was measured by various morphological and physiological indicators under salt stress, this work was complicated and time consuming, which need a long time and a large space and bring great difficulties to salt-tolerant breeding of rapeseed. With the development of molecular biology, molecular breeding technology is increasingly used in rapeseed germplasm innovation and improvement. In this study, many QTLs related to salt tolerance were identified, and a number of molecular markers linked to these major QTLs were obtained, such as SSR and IP markers associated with SPAD. These markers can be used to screen salt tolerant germplasm of rapeseed and cultivate salt-tolerant rapeseed. The utilization of these molecular markers combined with comprehensive trait observation will not only save time and space for breeders, but also the accuracy of selection will be increased substantially.

## Authors contributions

ZH was responsible for designing this study and drafting the manuscript; LL and JuD carried out the QTL mapping of salt tolerance; AX carried out gene cloning and expression; YZ, ZT, YL, and NZ carried out the data analysis and manuscript preparation; YW, XL, FL, BZ, MQ, and JaD collected important background information and provided assistance for data acquisition, data analysis and statistical analysis. All authors have read and approved the content of the manuscript.

### Conflict of interest statement

The authors declare that the research was conducted in the absence of any commercial or financial relationships that could be construed as a potential conflict of interest.

## Abbreviations

AFLP: Amplified Fragment Length Polymorphism
CIM: Composite interval mapping
CTAB: Cetyl trimethyl ammonium bromide
EC: Electrical conductivity
IP: Intron polymorphic
LFW: Leaf fresh weight
LGs: Linkage groups
LDW: Leaf dry weight
LOD: Log likelihood of the odds
ORF: Open reading frame
QTLs: Quantitative trait loci
RDW: Root dry weight
RL: Root length
SH: Seedling height
SOD: Superoxide dismutase
SP: Soluble protein
SPAD: Soil and Plant Analyzer Development
STR: Salt tolerance rating.
